# Metabolic syndrome among hypertensive patients at University of Gondar Hospital, North West Ethiopia: a cross sectional study

**DOI:** 10.1186/1471-2261-14-177

**Published:** 2014-12-06

**Authors:** Belaynesh Tachebele, Molla Abebe, Zelalem Addis, Nebiyu Mesfin

**Affiliations:** Hospital Laboratory, University of Gondar Hospital, Gondar, Ethiopia; Department of Clinical Chemistry, University of Gondar, School of Biomedical and Laboratory Sciences, Gondar, Ethiopia; Department of Medical Microbiology, University of Gondar, School of Biomedical and Laboratory Sciences, Gondar, Ethiopia; Department of Internal Medicine, University of Gondar, School of Medicine, Gondar, Ethiopia

**Keywords:** Hypertension, Metabolic syndrome, Cardiovascular risk, Dyslipidemia, Insulin resistance

## Abstract

**Background:**

Data is sparse concerning the magnitude of metabolic syndrome (MetS) in developing countries like Ethiopia whose major health problem had long been under-nutrition and infectious diseases rather than non-communicable diseases (NCDs) including hypertension, obesity and MetS. However, it is obvious that the NCDs are recently taking over and becoming the major health care concerns in the developing countries. This pattern could be partly explained by the nation’s sustained economical growth in the last few decades in addition to the increasing globalization related adoption of western lifestyle. The aim of this study was to assess the prevalence of metabolic syndrome and associated factors among hypertensive patients in North West Ethiopia.

**Method:**

A cross sectional study was conducted on 300 hypertensive individuals who get follow-up care at University of Gondar Hospital after diagnosed as hypertensive. The WHO STEP-wise approach to surveillance of NCD was used. Fasting blood glucose level, triglyceride and high density lipoprotein cholesterol were determined using standardized laboratory procedures.

**Result:**

The prevalence of metabolic syndrome was 40.7% and 39.3% according to the modified NCEP-ATP III and IDF criteria respectively. Low HDL-c was found to be the most frequently encountered (81.3%) component of MetS other than the hypertension. Being female was significantly associated with MetS (AOR = 4.34; 95% CI: 2.09, 8.99) using IDF and (AOR = 3.30; 95% CI: 1.66, 6.58) using NCEP-ATP III criteria. Abnormal BMI which included both high and low BMI was found to have significant association with MetS (AOR = 3.10; 95% CI: 1.73, 5.58) using IDF and (AOR = 1.84; 95% CI: 1.05, 3.22) as diagnosed using the NCEP-ATP III criteria.

**Conclusion:**

We recommend a comprehensive medical care approach to hypertensive patients to adequately assess and address the additional components of MetS which are known to potentiate the risks of cardiovascular diseases among hypertensive patients.

## Background

Hypertension and Metabolic syndrome (MetS) are highly prevalent diseases that present a global challenge [1, 2]. In 2000, approximately 1 billion people worldwide (26.4% of the adult population) were estimated to have hypertension and this is likely to increase to over 1.5 billion by 2025 as a result of aging population in many developed countries, and an increasing incidence of hypertension in developing countries [2]. In Ethiopia the prevalence of hypertension is estimated to be 35.2% [3]. It is also estimated that around 20-25% of the world’s adult population has metabolic syndrome and they are twice as likely to die from it; and they are three times more likely to have a heart attack or stroke compared with people without the syndrome [3, 4].

When hypertension and MetS components co-exist in an individual they potentiate one another leading to synergism that increase the total CVD risk [5]. Hypertension is one of the major manifestations of the group of clinical abnormalities that characterize metabolic syndrome found in 30 to 40% of hypertensive individuals [6]. Use of some antihypertensive agents like diuretics or β-adrenergic blocking agents may worsen insulin resistance and increase the risk of developing cardiovascular disease [7].

Even though there are different studies done on the prevalence of metabolic syndrome in different parts of the world [7–10], data regarding the situation in Africa especially Ethiopia is very sparse; and even the few reports on MetS in the area are not specifically done among hypertensive individuals [11, 12]. To the best of our knowledge, there are no reports on the prevalence of metabolic syndrome among hypertensive patients in Ethiopia particularly in the study area. Hence, the aim of this study was to determine the prevalence of metabolic syndrome among hypertensive patients and assess the associated factors.

## Methods

### Study setting and study population

This study was conducted in the University of Gondar hospital, from July 1 to August 31, 2013. The hospital has more than 500 beds capacity and serves as a tertiary level referral center for over five million people in and around Gondar town. This cross sectional study was conducted among hypertensive patients having follow-up care at the hypertension clinic of the hospital which is scheduled as a day weekly service at chronic illness clinic.

### Sample size and sampling technique

The sample size was estimated based on single population proportion formula using a confidence interval (CI) of 95% and a 31.2% previous prevalence of metabolic syndrome among hypertensive patients from Nigeria [13].


Where, n = sample size, p = proportion of hypertensive patients who may have metabolic syndrome and d = assumed marginal error. Based on the above formula and assumptions, the sample size was calculated to be 300. Then, every third participants were selected using systematic random sampling technique. There were a total of 3000 registered hypertension patients attending the clinic with an average follow-up interval of 2-3 month. All participants were with the age of greater than or equal to 18 years.

### Data collection

#### Data on the characteristics of study participants

The socio-demographic, behavioral, physical and clinical characteristics of the study participants were collected using an interview based structured questionnaire adopted from the WHO STEPS manual that was prepared for this purpose [14]. Data that were not captured by the interview were completed by assessing the medical records of the patients.

#### Anthropometric and blood pressure measurement

Anthropometric data (weight, height and waist circumference) were collected according to WHO STEPS manual [14]. Height was measured using stadiometer and weight was recorded after measuring the patient bare footed and wearing light clothes using a weight balance. During the height measurement, the participant’s shoes and any hats or hair ornaments were removed. The subject faces away from the wall with the heels together and the back as straight as possible. The head, shoulders, buttocks, and heels should be in contact with the vertical surface. With the subject looking straight ahead, the head projection is placed at the crown of the head. The participant steps away from the wall and the height measurement is recorded to the nearest 0.1 cm. From weight and height measurements, body mass index was calculated. Waist circumference (in centimeter) was measured at the midpoint between the lowermost rib and the iliac crest.

Blood pressure (BP) was measured using a standard adult arm cuff of mercury type sphygmomanometer after 10 min rest in the clinic by the nurses working in the chronic diseases follow up clinic. To improve the reliability of measurement two readings were taken with 1 min interval and the average of the two readings was recorded as the final BP of the patient. But if the difference between the two readings was greater than 5 mmHg, a third measurement was taken and the average of the three readings was recorded as the final BP of the patient [15].

### Laboratory investigation

Five milliliter of venous blood was collected from each patient in plane test tubes and serum was separated immediately. The extracted serum was investigated for High density lipoprotein-cholesterol (HDL-c), triglyceride and fasting glucose using Mindray BS-200 chemistry analyzer (Shenzen Mindray Bio-Medical electronics Co. Ltd) according to manufacturer’s instructions.

Triglycerides was determined by enzymatic hydrolysis with lipase (Human Gesellschaft fur Biochemica und Diagnostica mbH, Germany), HDL-c was determined by specific enzymatic method after eliminating chylomicrons, very low density lipoproteins and low density lipoproteins (Human Gesellschaft fur Biochemica und Diagnostica mbH, Germany) and glucose was determined by glucose oxidase method (LINEAR CHEMICALS S.L., Barcelona).

#### Definition of metabolic syndrome

Metabolic syndrome was defined according to modified NCEP-ATP III and IDF criteria.

#### IDF definition

In accordance with the IDF criteria, subjects were classified as having MetS if participants had abdominal obesity (defined as waist circumference of *≥*94 cm for men and *≥*80 cm women) plus two of any of the following risk factors: (1) raised TG level (*≥*150 mg/dL); (2) reduced HDLC (*<*40 mg/dL in males and *<*50 mg/dL in females); (3) raised blood pressure (systolic BP *≥*130 or diastolic BP *≥*85 mmHg) or treatment of previously diagnosed hypertension; (4) raised FG (*≥*100 mg/dL) [16].

#### Modified NCEP-ATP III definition

In accordance with the ATP III criteria, subjects were classified as having MetS if participants had three or more of the following risk factors: (1) abdominal obesity (waist circumference *>*102 cm in males and *>*88 cm in females); (2) hypertriglyceridemia (TG *≥*150 mg/dL); (3) reduced HDL-c (*<*40 mg/dL in males and *<*50 mg/dL in females); (4) high BP (*≥*130/85 mmHg); (5) FG (*≥*100 mg/dL) [17].

### Data analysis

Data was entered into EPI INFO computer software and exported to SPSS version 16 for analysis. Frequency distributions of socio-demographical, clinical and behavioral characteristics of subjects were explored. Continuous variables were expressed as mean *±* standard deviation or median (inter quartile range) and categorical variables were expressed as number (percentage). Univariate and multivariate binary logistic regression analysis was used to evaluate the differences in the distribution of categorical variables for study groups. P-value < 0.2 was used as a cutoff to include variables for multivariate binary logistic regression model. Student’s *t*-tests was used to evaluate differences in mean for study groups. The prevalence estimates for metabolic syndrome according to the two definitions was determined separately. In all cases P-values < 0.05 at 95% confidence level was considered statistically significant.

### Data quality control

Trained nurses working in the chronic diseases follow up clinic were involved in the collection of socio-demographic, behavioral, clinical and physical characteristics from the patients. The proper functioning of instruments, laboratory reagents, and technical performance was checked by using quality control samples. Standard operating procedures (SOPs) were followed starting from sample collection up to result reporting. All laboratory procedures were handled by laboratory technologists.

### Ethical considerations

Ethical clearance was obtained from School of Biomedical and Laboratory Sciences, University of Gondar. Before enrolling any of the eligible study participants; the purpose, benefits and risk of the study was clarified and discussed for each participant. Data was collected after informed consent was obtained and confidentiality of the information was kept by using codes. The results of the participants were communicated with the clinicians for better management of the patients.

## Results

### Sociodemographic and behavioral characteristics of the study participants

A total of 300 (115 male and 185 female) hypertensive patients, with a mean (±SD) age of 54 (±14) years, participated in this study. Majority, 78.3%, 56%, 41% of the study participants were urban dwellers, married and educationally unable to read and write respectively. Only 8.3% of the study participants had alcoholic drink at least once in the previous year and only 1% of the participants had a history of smoking. Palm oil was used by 55% of the study participants to prepare food. The 21.7% of the study participants had a day to day working habit full of vigorous intensity of activities which includes farming; long distance walking, laborious activities. Only 6% of the study participants have history of performing a purposely planned regular physical exercise (Table 1).

**Table 1:** **Sociodemographic and behavioral characteristics of the hypertensive patients at University of Gondar Hospital, July 1-August 31/2013**

Variable	Men (115)	Women (185)	P-value
	N (%)	N (%)	
Age			
< 30	9 (7.8)	11 (5.9)	0.001
30-50	30 (26.1)	88 (47.6)	
≥51	76 (66.1)	86 (46.5)	
Residence			
Urban	85 (73.9)	150 (81)	0.143
Rural	30 (26.1)	35 (18.9)	
Educational Status			
Unable to read and write	28 (24.3)	96 (51.9)	<0.001
Primary school	44 (38.3%)	44 (23.8%)	
Secondary school and above	43 (37.4%)	45 (24.3%)	
Marital Status			
Single	14 (12.2)	13 (7)	<0.001
Married	96 (83.5)	72 (38.9)	
Divorced	1 (0.87)	35 (19)	
Widowed	4 (3.5)	65 (35.1)	
Occupation			
Government employee	24 (20.9)	27 (14.6)	<0.001
Unemployed	36 (31.3)	122 (65.9)	
Self employee	55 (47.8)	36 (19.5)	
Monthly income (in ETB)^‡^			
≤ 600	61 (53.0)	121 (65.4)	0.048
601-1500	35 (30.4)	48 (25.9)	
≥1501	19 (16.5)	16 (8.6)	
Oil used in food preparation			
Palm oil	76 (66.1)	89 (48.1)	0.002
Cereal oil	39 (33.9)	96 (51.9)	
Work involving vigorous intensity of activity^£^			
Yes	30 (26.1)	35 (18.9)	0.143
No	85 (73.9)	150 (81.1)	
Regular physical exercise*			
Yes	10 (8.7)	8 (4.3)	0.121
No	105 (91.3)	177 (95.7)	

^‡^ETB = Ethiopian birr, is the formal currency of the country;

^£^Participants who daily walks for over one hour to go to work, or participants who are farmers or day laborers whose work doesn’t predominantly involve sitting;

^*^Participants who purposefully made any kind physical exercise for over thirty minute three times a week.

### Clinical features, biochemical values and physical characteristics of the study participants

From the study participants 21% reported family history of one or more of the chronic diseases (hypertension, diabetes mellitus and/or cardiac problem) and 94.7% started antihypertensive drug therapy. Among the patients who started treatment 42% were taking at least two types of antihypertensive drugs. The median (IQR) time since the initiation of treatment was 2 (0.4-4) years for men and 2 (0.5-6) years for women. Abnormal body mass index, which in this study includes both high and low BMI, was observed among 40% (121/300) of the study participants; women (45.4%) being more frequently affected as compared to their male (32.2%) counterparts. Women also showed higher mean concentrations of glucose (93.3 mg/dl) and HDL cholesterol (37.8 mg/dl). The median triglyceride values were 107 mg/dl for men and 115 mg/dl for women (Table 2).

**Table 2:** **Clinical features, biochemical values and physical characteristics of hypertensive patients at University of Gondar Hospital, July 1-August 31/2013**

Characteristics	Men (115)	Women (N = 185)	P-value
	n (%)	n (%)	
Family history of chronic diseases			
Yes	23 (20)	40 (21.6)	0.74
No	92 (80)	145 (78.4)	
Duration since hypertension diagnosis			
< 1 year	42 (36.5)	67 (36.2)	0.257
1-5 years	49 (42.6)	65 (35.1)	
≥6 years	24 (20.9)	53 (28.8)	
Anti-hypertensive treatment started			
Yes	110 (95.7)	174 (94.1)	0.549
No	5 (4.3)	11 (5.9)	
Duration since anti-hypertensive started			
< 1 year	43 (39.1)	78 (44.8)	0.029
1-5 years	49 (44.5)	52 (29.9)	
≥6 years	18 (16.4)	44 (25.3)	
Combination of hypertensive drugs			
0	5 (4.3)	11 (5.9)	0.435
1	44 (38.3)	83 (44.9)	
2	52 (45.2)	73 (39.5)	
3	14 (12.2)	18 (9.7)	
Body mass index (Kg/m^2^)			
Normal	78 (67.8)	101 (54.6)	0.023
Abnormal^£^	37 (32.2)	84 (45.4)	
	Mean (SD)	Mean (SD)	
Systolic blood pressure (mmHg)	134.7 (20.4)	136.6 (21.5)	0.866
Diastolic blood pressure (mmHg)	83.5 (11)	83.4 (11.6)	0.465
Glucose (mg/dl)	87.1 (20.4)	93.3 (28.1)	0.044
HDL cholesterol (mg/dl)	31.9 (12.2)	37.8 (12.5)	<0.001
Waist circumference (cm)	85.9 (11.2)	80.99 (12.6)	<0.01
	Median (IQR)	Median (IQR)	
Triglyceride (mg/dl)	107 (73, 134)	115 (83, 163)	0.0635*

*Data are median (inter-quartile range) since distribution was skewed, and test of significance was based on log-transformed values;

^£^Both high and low body mass index was merged together as abnormal BMI for analysis purpose.

### Prevalence of metabolic syndrome and frequency of metabolic syndrome components

The prevalence of metabolic syndrome was calculated based on the two criteria. According to the modified NCEP-ATP III criteria the overall prevalence of metabolic syndrome was 40.7%, women having high rate of metabolic syndrome, 46.5%, as compared to men, 31.3%. A relatively similar result was also obtained using the IDF criteria. Based on the IDF criteria 125 (41.7%) hypertensive patients had abdominal obesity among whom 118 (94.4%) fulfilled two additional metabolic syndrome components. Hence, the overall prevalence of MetS according to the IDF criteria was 39.3%; 24.3% among men and 48.6% among women. The frequency of metabolic syndrome components, other than the hypertension, according to the modified NCEP-ATP III criteria was 18.7%, 25.3%, 27.3% and 81.3% for abdominal obesity, elevated fasting glucose, elevated triglyceride and reduced HDL-c respectively. Higher prevalence of abdominal obesity and reduced HDL-c were observed among women than men. These values were similar applying IDF criteria except for abdominal obesity which the prevalence was 58.3% with significantly higher values among women (51.9%) compared to men (25.2%) (Table 3).

**Table 3:** **Frequency of metabolic syndrome (MetS) components other than elevated blood pressure (HTN) among hypertensive patients at University of Gondar Hospital, July 1- August 31/2013**

MetS component other than HTN	Overall frequency N = 300; n (%)	Men N = 115; n (%)	Women N = 185; n (%)	P-value
Abdominal obesity by NCEP-ATP III	56 (18.7)	9 (7.8)	47 (25.4)	<0.001
Abdominal obesity by IDF	125 (58.3)	29 (25.2)	96 (51.9)	<0.001
Elevated fasting glucose	76 (25.3)	23 (20)	53 (28.6)	0.094
Elevated triglyceride	82 (27.3)	26 (22.6)	56 (30.3)	0.148
Reduced HDL-c	244 (81.3)	86 (74.8)	158 (85.4)	0.022

According to the modified NCEP-ATP III criteria 53% men and 51.9% women had one additional metabolic syndrome component other than hypertension. Using the IDF criteria 43.5% of men and 34.6% of women had one additional MetS component other than elevated blood pressure (Table 4). The IDF criteria also showed that 13.9% men and 22.2% women had two metabolic syndrome components in addition to abdominal obesity.

**Table 4:** **Number of metabolic syndrome (MetS) components other than elevated blood pressure (HTN) by Gender according to the modified NCEP-ATP III criteria and the IDF criteria at University of Gondar Hospital, July 1- August 31/2013**

No. of MetS components other than HTN	Modified NCEP-ATP III criteria (%)		IDF criteria (%)	
	Men	Women	Men	Women
0	15.7	32	14.8	3.2
1	53	51.9	43.5	34.6
2	23.5	24.3	28.7	34.1
3	6.1	1.8	10.4	18.9
4	17.3	4.3	2.6	9.2

### Factors associated with prevalence of metabolic syndrome

According to the modified NCEP-ATP III criteria being female (COR = 1.91; 95% CI: 1.17, 3.12), being a government employees (COR = 3.60; 95% CI: 1.76, 7.39), being unable to read and write (COR = 0.36; 95% CI: 0.21, 0.64), having a monthly income of 601-1500 Ethiopian birr (COR = 1.84; 95% CI: 1.09, 3.13), having abnormal BMI (COR = 2.97; 95% CI: 1.84, 4.80) and using palm oil for food preparation (COR = 0.54; 95% CI: 0.34,0.86) were found to be significantly associated with the development of MetS. However, on the multivariate analysis the association was maintained for being female (AOR = 3.30; 95%CI: 1.66, 6.58), being unable to read and write (AOR = 0.30; 95%CI: 1.66, 6.58) and abnormal BMI (AOR = 1.84; 95% CI: 1.05, 3.22). Additionally, some variables that didn’t show significant association on the univariate analysis such as duration since hypertension diagnosis 1-5 years (AOR = 0.21; 95% CI: 0.06, 0.71), duration since hypertension diagnosis >5 years (AOR = 0.16; 95%CI: 0.03, 0.86), duration since on anti-hypertensive therapy 1-5 years (AOR = 5.04; 95% CI: 1.56, 16.33) and duration of anti-hypertensive therapy >5 years (AOR = 6.73; 95% CI: 1.26-36.00) were found to have significant association (Table 5).

**Table 5:** **Factors associated with metabolic syndrome (MetS) according to the modified NCEP-ATP III criteria among hypertensive patients at University of Gondar Hospital, July 1- August 31/2013**

Variables	MetS	No MetS	Crude OR (95% CI)	P-value	Adjusted OR (95% CI)	P-value
	N (%)	N (%)				
Sex						
Male	36 (12.0)	79 (26.3)	1		1	
*Female*	*86 (28.7)*	*99 (33.0)*	*1.91 (1.17,3.12)*	*< 0.01*	*3.30 (1.66, 6.58)*	*0.001*
Age						
< 30	6 (2.0)	14 (4.7)	1			
30-50	59 (19.7)	59 (19.7)	2.33 (0.84, 6.49)	0.10	1.94(0.60, 6.27)	0.27
≥51	57 (19.0)	105 (35.0)	1.27 (0.46, 3.48)	0.65	2.10(0.62, 7.12)	0.24
Educational status						
*Unable to read and write*	*40 (13.3)*	*84 (28.0)*	*0.36 (0.21, 0.64)*	*<0.001*	*0.30 (0.12, 0.74)*	*0.01*
Primary schools	32 (10.7)	56 (18.7)	0.43 (0.24, 0.80)	<0.01	0.55 (0.25, 1.21)	0.14
Secondary school or more	50 (16.7)	38 (12.7)	1		1	
Occupation						
Government employee	32 (10.7)	19 (6.3)	3.60 (1.76, 7.39)	< 0.001	2.14 (0.84, 5.45)	0.11
Unemployed	61 (20.3)	97 (32.3)	1.34 (0.78, 2.32)	0.29	0.96 (0.49, 1.85)	0.89
Self employed	29 (9.7)	62 (20.7)	1		1	
Monthly income in ETB^‡^						
≤ 600	63 (21.0)	119 (39.7)	1		1	
601-1500	41 (13.7)	42 (14.0)	1.84 (1.09, 3.13)	0.02	0.72 (0.28, 1.88)	0.51
≥1501	18 (6.0)	17 (5.7)	2.00 (0.96, 4.15)	0.06	0.93 (0.35, 2.42)	0.88
Oil used in food preparation						
Palm oil	56 (18.7)	109 (36.3)	0.54 (0.34, 0.86)	<0.01	0.61 (0.36, 1.06)	0.08
Cereal oil	66 (22.0)	69 (23.0)	1			
Regular physical exercise^*^						
Yes	10 (3.3)	8 (2.7)	1			
No	112 (37.3)	170 (56.7)	0.53(0.20, 1.38)	0.19	2.15 (0.68, 6.86)	0.20
Duration since hypertension diagnosis						
< 1 year	38 (12.7)	71 (23.7)	1			
*1-5 years*	*47 (15.7)*	*67 (23.3)*	*1.31 (0.76, 2.26)*	*0.33*	*0.21 (0.06, 0.71)*	*0.01*
*≥6 years*	*37 (12.3)*	*40 (13.3)*	*1.73 (0.95, 3.14)*	*0.07*	*0.16 (0.03, 0.86)*	*0.03*
Duration since anti-hypertensive started						
< 1 year	43 (15.1)	78 (27.5)	1			
*1-5 years*	*45 (15.8)*	*56 (19.7)*	*1.55 (0.85, 2.50)*	*0.17*	*5.04 (1.56, 16.33)*	*0.01*
*≥6 years*	*31 (10.9)*	*31 (10.9)*	*1.81 (0.97, 3.39)*	*0.06*	*6.73 (1.26, 36.00)*	*0.03*
Body Mass Index (BMI)						
Normal	54 (18.0)	125 (41.7)	1		1	
*Abnormal* ^*£*^	*68 (22.7)*	*53 (17.7)*	*2.97 (1.84, 4.80)*	*<0.001*	*1.84 (1.05, 3.22)*	*0.03*

^‡^ETB = Ethiopian birr, is the formal currency of the country; COR = crude odds ratio; AOR = adjusted odds ratio; CI = confidence interval.

^*^Participants who purposefully made any kind physical exercise for over thirty minute three times a week;

^£^Both high and low body mass index was merged together as abnormal BMI for analysis purpose;

P-value < 0.2 was used to include the variables for adjusting in the multivariate binary logistic regression.

Similarly, applying the IDF criteria for MetS, the univariate analysis showed that being female (COR = 3.01; 95% CI: 1.80, 5.03), being a government employees (COR = 2.96; 95% CI: 1.42, 6.18), having abnormal BMI (COR = 4.40; 95% CI: 2.68, 7.21), having an educational status of less than primary school (COR = 0.38; 95% CI: 0.21, 0.71), living in the rural area (COR = 0 .27; 95% CI: 0.14, 0.53), duration since hypertension diagnosis > 5 years (COR = 2.51; 95% CI: 1.37, 4.60) and duration since ant-hypertensive therapy > 5 years (COR = 2.24; 95% CI: 1.20, 4.20) were found to be significantly associated with the development of MetS. However, the multivariate analysis showed that being female (AOR = 4.34; 95% CI: 2.09, 8.99), having an educational status of primary school (AOR = 0.40; 95% CI: 0.17, 0.93), being unable to read and write (AOR = 0.39; 95% CI: 0.15, 1.02) and abnormal BMI (AOR = 3.10; 95% CI: 1.73, 5.58) have maintained the significant association with MetS (Table 6).

**Table 6:** **Factors associated with metabolic syndrome (MetS) according to the IDF criteria among hypertensive patients at University of Gondar Hospital, July 1- August 31/2013**

Variables	MetS	No MetS	Crude OR (95% CI)	P-value	Adjusted OR (95% CI)	P-value
Sex						
Male	28 (9.3)	87 (29)	1		1	
*Female*	*91(30.3)*	*94 (31.3)*	*3.01 (1.80, 5.03)*	*<0.001*	*4.34(2.09, 8.99)*	*<0.001*
Age						
<30	5 (1.7)	15 (5)	1			
30-50	50 (16.7)	68(22.7)	2.21 (0.75, 6.47)	0.15	0.74(0.20, 2.71)	0.65
>51	64 (21.3)	98 (32.7)	1.96 (0.68, 5.66)	0.21	1.27(0.34, 4.77)	0.73
Residence						
Urban	107 (35.7)	128 (42.7)	1			
Rural	12 (4)	53 (17.7)	0.27 (0.14, 0.53)	<0.001	0.60(0.25, 1.42)	0.24
Educational status						
*Unable to read and write*	*47 (15.7)*	*77 (25.7)*	*0.56(0.32, 0.97)*	*0.04*	*0.39(0.15, 1.02)*	*0.05*
*Primary schools*	*26 (8.7)*	*62 (20.7)*	*0.38 (0.21, 0.71)*	*0.02*	*0.40(0.17, 0.93)*	*0.03*
Secondary school or more	46 (15.3)	42 (14)	1		1	
Occupation						
Government employee	24 (8)	27 (9)	2.96 (1.42, 6.18)	0.004	1.10(0.41, 2.91)	0.85
Unemployed	74 (24.7)	84 (28)	2.94 (1.65, 5.24)	<0.001	1.64(0.78, 3.43)	0.19
Self employed	21 (7)	70 (23.3)	1		1	
Monthly income in ETB^‡^						
≤ 600	66 (22)	116 (38.7)	1			
601-1500	39(13)	44 (14.7)	1.56 (0.92, 2.64)	0.10	1.57(0.81, 3.06)	0.19
≥1501	14 (4.7)	21 (7)	1.17 (0.56, 2.46)	0.68	1.16(0.40, 3.34)	0.79
Oil used in food preparation					1	
Palm oil	59 (19.7)	106 (35.3)	0.70(0.44, 1.11)	0.13	0.88(0.49, 1.56)	0.66
Cereal oil	60 (20)	75 (25)	1		1	
Duration since hypertension diagnosis						
< 1 year	34 (11.3)	75 (25)	1		1	
1-5 years	44 (14.7)	70 (23.3)	1.39 (0.80, 2.41)	0.25	0.59(0.19, 1.82)	0.36
≥6 years	41 (13.7)	36 (12)	2.51 (1.37, 4.60)	0.003	1.36(0.29, 6.32)	0.70
Duration since anti-hypertensive started						
< 1 year	39 (13.7)	82 (28.9)	1		1	
1-5 years	43 (15.1)	58 (20.4)	1.56(0.90, 2.70)	0.11	2.45(0.83, 7.23)	0.10
≥6 years	32 (11.3)	30 (10.6)	2.24 (1.20, 4.20)	0.01	1.11(0.24, 5.21)	0.90
Body Mass Index (BMI)						
Normal	46 (15.3)	133 (44.3)	1		1	
*Abnormal* ^*£*^	*73 (24.3)*	*48 (16)*	*4.40(2.68, 7.21)*	*<0.001*	*3.10(1.73, 5.58)*	*<0.001*

^‡^ETB = Ethiopian birr, is the formal currency of the country; COR = crude odds ratio; AOR = adjusted odds ratio; CI = confidence interval.

^£^Both high and low body mass index was merged together as abnormal BMI for analysis purpose;

P-value < 0.2 was used to include the variables for adjusting in the multivariate binary logistic regression.

## Discussion

This study showed high prevalence of metabolic syndrome among hypertensive patients attending followup at University of Gondar Hospital. Using the modified NCEP-ATP III criteria the prevalence of metabolic syndrome was found to be 40.7% which is relatively similar to the prevalence obtained according to the IDF criteria (39.3%). Moreover, females were found to have a higher prevalence of metabolic syndrome (NCEP-ATP III = 46.5%; IDF = 48.6%) as compared to men (NCEP-ATP III = 31.3%; IDF = 24.3%). Though not statistically significant, increased occurrence of metabolic syndrome was also observed in the subjects aged ≥50 years.

A study from Nigeria has reported a metabolic syndrome prevalence of 31.2% among newly diagnosed hypertensive patients according to the NCEP-ATP III criteria and a study from Kuwait reported a prevalence of 34% [5, 9]. These reports are slightly lower than the finding in our study; this might be explained by the fact that the Nigerian study included only newly diagnosed hypertensive patients who didn’t start anti-hypertensive treatment. And, this could underscore the magnitude of the MetS when compared to a study that includes chronically hypertensive patients. Moreover, there could be socio-demographic, lifestyle and genetic differences between our population and the population of Kuwait. On the other hand, slightly higher and lower prevalence of MetS was reported from Nigeria (45.6%), Taiwan (31.3%), and other parts of the world [13, 18–20]. And, these reports are comparable to our study.

The frequency of metabolic syndrome components in this study was 18.7%, 25.3%, 27.3% and 81.3% for abdominal obesity, elevated fasting glucose, elevated triglyceride and reduced HDL-c respectively. All these values, except for reduced HDL-c, were lower than a study conducted in Jordan [20]. This might be explained by the difference in lifestyle and socioeconomic status of the two populations which is reflected by high rates (86.1%) of obese individuals in the Jordan study. In the contrary, the report in the current study was higher than a study conducted in Nigeria that showed the percentage of individuals with MetS were, 40.4% for men and 38.9% for women according to the modified NCEP-ATP III criteria [5]. This may be due to the fact that only newly diagnosed individuals were included in the Nigerian study.

Concordant to our finding, the association between sex and metabolic syndrome was reported in other studies [7, 21]. Individuals with abnormal BMI were found to have a significant association with MetS as compared to those with normal BMI. In this study both low and high BMI values were merged as abnormal BMI for the purpose of analysis. Literatures are now coming out indicating that low BMI and malnutrition may contribute to low HDL-c; [22] high BMI is known to be associated with low HDL-c and high waist circumference, a cumulative effect of which could lead to high prevalence of MetS among individuals with abnormally high or low BMI [22]. The NCEP-ATP III criteria showed significant association between the occurrence of MetS and the duration since hypertension diagnosis and treatment. This could be partly explained by the possibility of having more components of MetS in a chronically hypertensive patient compared to the newly diagnosed ones. Moreover, though specific anti-hypertensive drug type stratification and analysis was not done, it may indicate that some of the antihypertensive drugs like diuretics or β-adrenergic blocking agents could contribute to insulin resistance and hence to higher rates of MetS [7].

Our study has indicated that having an educational status of less than of less than primary school level has significant association with the occurrence of Mets. This could be explained by the fact that the lifestyle, type of work and even place of residence could be affected by the level of education which may also indirectly impact the risk and occurrence of MetS. This could also partly explain the effect of income on the occurrence of MetS.

Studies from Nigeria [5] and Kuwait [9] indicated the presence of an association between the age of hypertensive patients and metabolic syndrome prevalence. However in the current study age didn’t show any statistically significant association. This may be due to the inclusion of highly susceptible study subjects in the Kuwait and Nigerian studies.

Due to the cross sectional nature of the study, temporal relations could not be established between metabolic syndrome and the associated factors. Moreover, only two definitions were used to assess the prevalence of MetS; a different prevalence rate could have been observed if other metabolic syndrome definition like the WHO definition were used. Despite these limitations this study ultimately adds to the limited data on metabolic syndrome in sub-Saharan Africa, particularly in the study area.

## Conclusion

The prevalence of metabolic syndrome among hypertensive patients is high, with low HDL-c being the most commonly encountered abnormality. Being female, having abnormally high or low BMI and having less than primary school educational status were found to have significant association with the occurrence of MetS. Majority of the hypertensive individuals were at a high risk of developing MetS as they have one more very common additional risk factor (low HDL-c). Therefore, all hypertensive patients should be aggressively screened for the presence of additional cardiovascular risk factors that constitute the metabolic syndrome. Management of hypertensive patients in the presence of an additional component of metabolic syndrome should be comprehensive enough to address the increased risk of cardiovascular and diabetes related events. Moreover, further in depth studies should be conducted to assess predictors of MetS and the performance of the health care system in order to design, and implement a cost-effective and comprehensive patient care approach for hypertensive patients.

## Abbreviations

ATP III: Adult treatment panel
BMI: Body mass index
CVD: Cardiovascular diseases
HDL-c: High density lipoprotein cholesterol, IDF, International diabetics federation
MetS: Metabolic syndrome
NCEP: National cholesterol education program
NCDs: Non-communicable diseases
WHO: World Health Organization.

## References

[1] Cornier MA, Dabelea D, Hernandez TL, Lindstrom RC, Steig AJ, Stob NR, Van Pelt RE, Wang H, Eckel RH (2008). The metabolic syndrome. Endocr Rev.

[2] Kearney PM, Whelton M, Reynolds K, Muntner P, Whelton PK, He J (2005). Global burden of hypertension: analysis of worldwide data. Lancet.

[3] World Health Organization (WHO) (2011). Non-Communicable Diseases Country Profile.

[4] International diabetes federation (IDF) (2006). The IDF consensus worldwide definition of the metabolic syndrome.

[5] Osuji CU, Omejua EG (2012). Prevalence and characteristics of the metabolic syndrome among newly diagnosed hypertensive patients. Indian J Endocrinol Metab.

[6] Marchi-Alves LM, Rigotti AR, Nogueira MS, Cesarino CB, de Godoy S (2012). Metabolic syndrome components in arterial hypertension. S Rev Esc Enferm USP.

[7] Hsu CN, Chen YC, Wang TD (2005). Prevalence and characteristics of the metabolic syndrome in Chinese hypertensive patients: a hospital-based observation. Acta Cardiol Sin.

[8] Hilgers KF, Mann JF (2008). The choice of antihypertensive therapy in patients with the metabolic syndrome–time to change recommendations?. Nephrol Dial Transplant.

[9] Sorkhou EI, Al-Qallaf B, Al-Namash HA, Ben-Nakhi A, Al-Batish MM, Habiba SA (2004). Prevalence of metabolic syndrome among hypertensive patients attending a primary care clinic in Kuwait. Med Princ Pract.

[10] Pierdomenico SD, Lapenna D, Di Tommaso R, Di Carlo S, Caldarella MP, Neri M, Mezzetti A, Cuccurullo F (2007). Prognostic relevance of metabolic syndrome in hypertensive patients at low-to-medium risk. Am J Hypertens.

[11] Tran A, Gelaye B, Girma B, Lemma S, Berhane Y, Bekele T, Khali A, Williams MA (2011). Prevalence of metabolic syndrome among working adults in Ethiopia. Int J Hypertens.

[12] Berhane T, Yami A, Alemseged F, Yemane T, Hamza L, Kassim M, Deribe K (2012). Prevalence of lipodystrophy and metabolic syndrome among HIV positive individuals on Highly Active Anti-Retroviral treatment in Jimma, South West Ethiopia. Pan Afr Med J.

[13] Su CH, Fang CY, Chen JS, Po HL, Chou LP, Chiang CY, Ueng KC (2011). Prevalence of metabolic syndrome and its relationship with cardiovascular disease among hypertensive patients 55-80 years of age. Acta Cardiol Sin.

[14] World Health Organization (WHO) (2010). Chronic diseases and health promotion: Stepwise approach to surveillance (STEPS).

[15] Lemogoum D, Seedat YK, Mabadeje AF, Mendis S, Bovet P, Onwubere B, Blackett KN, Lenfant C, Kabangu JR, Block P, Belhocine M, Degaute JP (2003). Recommendations for prevention, diagnosis and management of hypertension and cardiovascular risk factors in sub-Saharan Africa. J Hypertens.

[16] Stern MP, Williams K, González-Villalpando C, Hunt KJ, Haffner SM (2004). Does the metabolic syndrome improve identification of individuals at risk of type 2 diabetes and/or cardiovascular disease?. Diab Care.

[17] Alberti KG, Eckel RH, Grundy SM, Zimmet PZ, Cleeman JI, Donato KA, Fruchart JC, James WP, Loria CM, Smith SC (2009). Harmonizing the metabolic syndrome: a joint interim statement of the International Diabetes Federation Task Force on Epidemiology and Prevention; National Heart, Lung, and Blood Institute; American Heart Association; World Heart Federation; International Atherosclerosis Society; and International Association for the Study of Obesity. Circulation.

[18] Ogbu ISI, Ugwuja EI (2012). Metabolic syndrome in hypertensive nigerians: risk factor analysis. IOSR J Pharm Biol Sci.

[19] Lioudaki E, Vrentzos GE, Mavrogeni H, Zeniodi MH, Ganotakis ES, Mikhailidis DP, Papadakis JA (2012). Prevalence of metabolic syndrome according to different definitions in a hypertensive population. Angiology.

[20] Yasein N, Ahmad M, Matrook F, Nasir L, Froelicher ES (2010). Metabolic syndrome in patients with hypertension attending a family practice clinic in Jordan. East Mediterr Health J.

[21] Shahbazian H, Latifi SM, Jalali MT, Shahbazian H, Amani R, Nikhoo A, Aleali AM (2013). Metabolic syndrome and its correlated factors in an urban population in South West of Iran. J Diab Metab Disord.

[22] Delisle H, Ntandou G, Després JP (2013). At-risk serum cholesterol profile at both ends of the nutrition spectrum in West African adults? The Benin Study. Nutrients.

[CR23] The pre-publication history for this paper can be accessed here:http://www.biomedcentral.com/1471-2261/14/177/prepub

